# Loneliness in young people: a multilevel exploration of social ecological influences and geographic variation

**DOI:** 10.1093/pubmed/fdab402

**Published:** 2022-01-07

**Authors:** J Marquez, C Goodfellow, D Hardoon, J Inchley, A H Leyland, P Qualter, S A Simpson, E Long

**Affiliations:** MRC/CSO Social and Public Health Sciences Unit, University of Glasgow, Glasgow G3 7HR UK; MRC/CSO Social and Public Health Sciences Unit, University of Glasgow, Glasgow G3 7HR UK; What Works Centre for Wellbeing, London SW1H 9EA, UK; MRC/CSO Social and Public Health Sciences Unit, University of Glasgow, Glasgow G3 7HR UK; MRC/CSO Social and Public Health Sciences Unit, University of Glasgow, Glasgow G3 7HR UK; University of Manchester, Manchester Institute of Education, Manchester M13 9PL, UK; MRC/CSO Social and Public Health Sciences Unit, University of Glasgow, Glasgow G3 7HR UK; MRC/CSO Social and Public Health Sciences Unit, University of Glasgow, Glasgow G3 7HR UK

**Keywords:** geographical, loneliness, multilevel modelling, social ecological, young people

## Abstract

**Background:**

Loneliness is a growing public health concern, yet little is known about loneliness in young people. The current study aimed to identify social ecological factors related to loneliness and examine the extent to which geographic region may account for differences in loneliness.

**Methods:**

The data come from a cross-sectional sample of 6503 young people living in the UK. Loneliness was measured using the UCLA 3-item scale. Bivariate analyses were used to test associations between each predictor and loneliness. Multilevel models were used to identify key social ecological factors related to loneliness, and the extent to which loneliness may vary across geographic regions (local authority districts).

**Results:**

Sociodemographic, social, health and well-being, and community factors were found to be associated with loneliness. Geographic region was associated with 5–8% of the variation in loneliness. The effect of gender, sexual orientation and minority ethnic background on loneliness differed across regions.

**Conclusions:**

This is the first study to highlight modifiable social and community factors related to youth loneliness, and individual vulnerabilities, such as poor mental well-being. Results related to geographic differences suggest that local-level initiatives may be most appropriate in tackling loneliness, rather than wider, less contextualized national efforts.

## Introduction

Loneliness is a key public health concern in the UK,^1^ with known associations with poorer physical health,^2^^,^^3^ increased use of health services^4^ and early mortality.^5^ Loneliness is commonly defined as the subjective emotional experience of an absence of desired social relationships.^6^^,^^7^ Recent research demonstrates that loneliness is a pervasive problem among young people^8^^,^^9^ with survey data indicating that those aged 16–24 years are 27% more likely to report loneliness than those aged 75 years or older,^9^ and young people were especially at risk of loneliness during the COVID-19 pandemic.^10^

However, the majority of research examining loneliness is among older adults. Studies that focus on younger adults (aged 65 years and younger) tend to study a broad age range, resulting in a lack of research which examines loneliness among young people specifically.^11^^,^^12^ Yet, loneliness in young people may be particularly detrimental, as loneliness is likely to be compounded by stigmatizing responses from peers due to social norms that favour connectedness.^13^^,^^14^

Where research does exist, studies demonstrate that loneliness among young people is associated with poorer mental health, higher levels of risk behaviours, (e.g. smoking),^15^ increased risk for disability and greater risk of unemployment in adulthood.^16^ Additionally, evidence suggests that the effects of loneliness can accumulate across the lifespan,^17–19^ highlighting the importance of early intervention strategies, and identification of risk factors for loneliness among young people.

Using a social ecological perspective^20^^,^^21^ loneliness is the product of multiple, interdependent domains of influence (e.g. individual, social, wider environmental factors). Consequently, to identify appropriate targets for public health interventions aimed at alleviating loneliness, it is critical for research to examine the comprehensive, interacting, set of risk factors that span the social ecological spectrum. Importantly, although individual sociodemographic factors may highlight certain vulnerabilities to loneliness, they tend to be unmalleable (e.g. gender), whereas social and community factors can be more easily integrated into prevention efforts for targeted populations.

Furthermore, while it is imperative to identify predictors of loneliness, we must also understand how those risks vary in different settings. Evidence from mid and older adult populations demonstrate differences in loneliness across geographic regions.^22^^,^^23^ Although some research has examined the impact of community-level factors on youth loneliness, such as rural versus urban environments,^24^ or neighbourhood deprivation,^15^ no study has investigated whether loneliness differs among young people in the UK based on geographic region. Consequently, it is currently unknown if there are differences across geographic areas in the strength of risk or protective factors for loneliness, or if individual differences in loneliness can be accounted for by the geographic region in which young people live.

As such, this is the first study to employ a multilevel modelling approach to identify social–ecological factors related to loneliness among young people in the UK, and to investigate whether geographic region (assessed in our study, via participant’s local authority (LA) district) accounts for differences in youth loneliness. This contextualized approach provides a more nuanced understanding of the mechanisms surrounding loneliness in young people, providing novel targets for public health interventions.

Specifically, this study explores the following research questions:

1. What are the key sociodemographic, social, health and well-being, and community factors associated with loneliness in young people?
2. Does geographic region explain variation in youth loneliness?
3. Are individual-level risk or protective factors for loneliness stronger in some geographic regions than others?

## Methods and data

### Participants

We use cross-sectional data from Understanding Society collected across the UK between 2017 and 2019. Understanding Society is a household panel survey of more than 40 000 households that began in 2009.^25^^,^^26^ Data on loneliness have not been routinely collected in Understanding Society, thus the current study utilizes the most recent loneliness data, captured at Wave 9. The sample consists of 6503 young people aged 16–24 years, distributed across 379 geographic regions.

### Measures

Framed by social ecological theory^21^ the study investigated potential predictors of loneliness across four domains: sociodemographics, health and well-being, social relationships, and community environment, resulting in 24 risk and protective factors. The full list of variables is described in Table 1 and summarized below.

**Table 1:** Descriptive statistics

Variable	Descriptive statistics	% of missing data
Loneliness (3-item UCLA scale)	Mean = 1.69; S.D. = 1.85; median = 1; Min = 0 (least lonely); Max = 6 (most lonely)	8.66%
Age	Mean = 19.81; S.D. = 2.58; Min = 16; Max = 24	0.00%
Gender	Male = 44.50%; female = 55.50%	0.00%
Sexual orientation (binary)	Heterosexual = 91.44%; not heterosexual = 8.56%	12.09%
Sexual orientation (categorical)	Heterosexual = 91.44%; gay/lesbian = 1.94%; bisexual = 5.44%; other = 1.17%	12.09%
Country	England = 80.27%; Wales = 5.34%; Scotland = 6.86%; N.Ireland = 7.53%	0.00%
Living in urban or rural area	Urban = 81.22%; rural = 18.78%	0.00%
Minority ethnic group	White British = 63.86%; minority ethnic group = 36.14%	0.00%
Belongs to religion	Does not belong to a religion = 56.72%; belongs to a religion = 43.28%	25.40%
Subjective financial situation	Mean = 3.87; S.D. = 0.97; Min = 1 (finding it very difficult); Max = 5 (living comfortably)	6.49%
Self-reported health	Mean = 3.66; S.D. = 0.97; Min = 1 (poor); Max = 5 (excellent)	7.89%
Mental well-being (GHQ)	Mean = 11.82; S.D. = 6.85; Min = 0 (excellent); Max = 36 (poor)	9.96%
Has a long-standing illness or disability	Yes = 17.64%; no = 82.36%	0.35%
Life satisfaction	Mean = 5.10; S.D. = 1.53; Min = 1 (completely dissatisfied); Max = 7 (completely satisfied)	8.50%
Hours interacting with friends on social media	Mean = 3.19; S.D. = 1.05; Min = 1 (none); Max = 5 (7 h or more)	13.79%
Going out socially/visiting friends when feeling like it	Yes = 85.57; no = 14.43%	4.84%
Number of close friends	Mean = 5.14; S.D. = 1.05; Min = 0; Max = 100	6.72%
Number of friends similar age	Mean = 3.28; S.D. = 0.89; Min = 1 (less than half); Max = 4 (all similar)	6.60%
Number of friends similar race	Mean = 2.93; S.D. = 0.97; Min = 1 less than half); Max = 4 (all similar)	6.98%
Number of friends living same area	Mean = 3.23; S.D. = 1.29; Min = 1 (none); Max = 5 (all)	6.78%
Perceived neighbourhood quality	Mean = 20.51; S.D. = 5.73; Min = 0 (lowest quality); Max = 28 (highest quality)	13.07%
Sense of belonging to neighbourhood	Mean = 3.52; S.D. = 1.05; Min = 1 (strongly disagree); Max = 5 (strongly agree)	8.80%
Local friends mean a lot	Mean = 3.24; S.D. = 1.13; Min = 1 (strongly disagree); Max = 5 (strongly agree)	8.87%
Similar to others in neighbourhood	Mean = 3.02; S.D. = 1.21; Min = 1 (strongly disagree); Max = 5 (strongly agree)	8.95%
Talk regularly to neighbourhood	Mean = 3.05; S.D. = 1.29; Min = 1 (strongly disagree); Max = 5 (strongly agree)	8.86%
Community type	Blue collar communities = 17.17%; city living = 3.41%; countryside = 9.37%; prospering suburbs = 17.54%; constrained by circumstances = 7.05%; typical traits = 15.76%; multicultural = 29.69%	0.00%
Geographic region (local authority district)	379 different geographic regions in the UK. Population range: 2224 (Isles of Scilly)—1 141 816 (Birmingham). Mean population: 176 245	0.00%

*n* = 6503. S.D. = standard deviation.

#### Loneliness

Loneliness was measured using the 3-item UCLA-loneliness scale, indicating how often (0 = ‘hardly ever or never’, 1 = ‘some of the time’ or 2 = ‘often’) the respondent felt a lack of companionship, left out and isolated. The 3-item UCLA scale is frequently used in population studies and is well validated.^27^ Responses to the three items were summed, creating a variable ranging from 0 to 6. In line with previous use of this scale,^28–30^ we considered loneliness as a continuous outcome.

#### Sociodemographic variables

The study controlled for a range of sociodemographic characteristics provided by participants. Age was recorded as age at time of survey completion. Gender was a binary indicator (male/female). Sexual orientation included heterosexual, gay or lesbian, bisexual, and other. Ethnic group was a binary indicator of White British or non-White British. The study included a binary measure of belonging to a religion, and a 5-point scale of subjective financial situation. Participants’ country of residence and whether they lived in an urban or rural location were also included.

#### Health and well-being variables

Self-reported health was measured on a 5-point scale, with higher scores indicating better health. The 12-item General Health Questionnaire^31^ was used to measure mental well-being, with higher scores indicating poorer mental well-being. A binary indicator of having a long-standing illness, and a single-item, 7-point measure of life satisfaction, with higher scores indicating higher satisfaction were included.

#### Social relationship variables

Participants indicated their number of close friends; the number of friends of similar age and race; and number of friends living in the same geographic area. A binary measure of going out socially with friends, and a 5-point scale indicating hours spent interacting with friends on social media were also included.

#### Community variables

Perceived neighbourhood quality was measured using a sum score from nine items related to characteristics of the neighbourhood (e.g. ‘extent of graffiti on walls’; Cronbach’s *α* = 0.901). Belonging to the neighbourhood; being similar to others in the neighbourhood; talking regularly with neighbours; and having local friends were each measured on a 5-point scale, with higher scores indicating higher agreement. Community type was measured using the Census 2001 Output Area Classification (OAC) included within the dataset, resulting in seven classifications^32^ (blue collar communities; city living; countryside; prospering suburbs; constrained by circumstances; typical traits; and multicultural).

#### Geographic region

Geographic region was measured by the LA district of the participant, covering a total of 379 LA districts in the UK. LAs are subnational divisions representing local governments across the four UK nations. LAs vary in size, and populations for LAs included in our dataset range from 2224 to over one million people.

For clarity, community variables refer to subjective assessment of one’s neighbourhood, as well as community type according to OAC. Geographic region refers to the 379 LAs. Within the multilevel modelling approach described subsequently, geographic region is the Level 2 grouping variable, whereas community factors are measured at Level 1 alongside the remaining social ecological domains.

### Analyses

Preliminary bivariate analyses were used to test the association between loneliness and each of the 24 risk and protective factors, unadjusted for the other covariates. Our loneliness variable was moderately skewed (skewness = 0.7; kurtosis = 2.6), which represents a mild violation of the assumption of normal distribution required in multilevel analyses. We used a series of nested multilevel models to answer our research questions. Multilevel techniques are well-suited for the current study, as they partition variation in loneliness attributable to differences between individuals (Level 1), as well as differences between geographic regions (Level 2).^33^

We first tested a null-model (Model 1) to determine whether there were significant between-region differences in loneliness scores, and hence, methods from multilevel modelling would be required. A likelihood-ratio test (LRT) was used to compare a model with a multilevel structure (e.g. respondents nested within regions) to a single-level linear regression model. We then ran a series of models (Model 2–Model 5) incorporating sociodemographic, health and well-being, social, and community variables sequentially, while accounting for geographic region. We estimated an additional full-model (Model 6) using a categorical version of sexual orientation (i.e. heterosexual, lesbian/gay, bisexual, other) rather than a binary one to investigate differences in loneliness between different minority sexual orientation groups. Lastly, we tested a series of random-slopes models to determine whether the associations between explanatory variables and loneliness differed across regions. Multiple imputations were used to account for missing data. We used a multivariate normal regression approach, which employs an iterative Markov chain Monte Carlo method to impute missing values. We performed 20 imputations of the data set. All analyses were conducted using Stata 15.^34^

## Results

### Descriptive statistics

Table 1 displays the descriptive statistics of the sample. On average, participants were ~19 years old and scored on the lower end of the loneliness scale (1.68, range 0–6). Approximately 55% of the sample were female, and the majority of participants resided in England.

### Bivariate and multilevel models

Table 2 displays the results for the bivariate and multilevel models. Results from the LRT in the null multilevel model (Model 1) demonstrated strong evidence of geographic region effects (on 1 d.f., LR > 16822.72), and across all models, between 5.20% and 7.56% of the variation in loneliness was associated with geographic region.

**Table 2:** Output from bivariate model and multilevel models which control for variation at the geographic level

Variables	Bivariate analysis		Model 1 (null model)		Model 2		Model 3		Model 4		Model 5 (full model A)		Model 6 (full model B)	
	*b*	SE	*b*	SE	*b*	SE	*b*	SE	*b*	SE	*b*	SE	*b*	SE
**Fixed:**														
Constant			1.760^*^^*^^*^	(0.037)	3.489^*^^*^^*^	(0.206)	2.542^*^^*^^*^	(0.214)	3.637^*^^*^^*^	(0.252)	4.158^*^^*^^*^	(0.271)	4.131^*^^*^^*^	(0.289)
*Sociodemographic*														
Age: 16–24	−0.003	(0.009)			−0.018^*^^*^	(0.009)	−0.016^*^^*^	(0.007)	−0.021^*^^*^^*^	(0.007)	−0.025^*^^*^^*^	(0.007)	−0.025^*^^*^^*^	(0.008)
Gender	0.279^*^^*^^*^	(0.045)			0.253^*^^*^^*^	(0.044)	−0.038	(0.039)	−0.041	(0.038)	−0.023	(0.039)	−0.023	(0.038)
Sexual orientation (binary): not heterosexual	1.159^*^^*^^*^	(0.081)			0.973^*^^*^^*^	(0.080)	0.477^*^^*^^*^	(0.069)	0.437^*^^*^^*^	(0.069)	0.392^*^^*^^*^	(0.069)		
Sexual orient. (cat.) (ref. hetero.): gay/lesbian	0.855^*^^*^^*^	(0.169)											0.404^*^^*^^*^	(0.137)
Sexual orient. (cat.) (ref. hetero.): bisexual	1.217^*^^*^^*^	(0.101)											0.309^*^^*^^*^	(0.085)
Sexual orient. (cat.) (ref. hetero.): other	1.527^*^^*^^*^	(0.226)											0.820^*^^*^^*^	(0.174)
Country (ref. England): Wales	−0.109	(0.101)			−0.294^*^^*^	(0.145)	−0.295^*^^*^	(0.116)	−0.244^*^^*^	(0.118)	−0.191^*^	(0.115)	−0.191^*^	(0.115)
Country (ref. England): Scotland	0.011	(0.089)			−0.160	(0.128)	−0.145	(0.102)	−0.105	(0.104)	−0.051	(0.102)	−0.055	(0.103)
Country (ref. England): Northern Ireland	−0.206^*^^*^	(0.088)			−0.182	(0.164)	−0.031	(0.127)	−0.003	(0.132)	0.073	(0.129)	0.081	(0.131)
Living in urban or rural area	0.006	(0.058)			−0.063	(0.068)	−0.014	(0.056)	−0.032	(0.056)	0.008	(0.067)	0.011	(0.068)
Minority ethnic group (non-White British)	−0.330^*^^*^^*^	(0.049)			−0.191^*^^*^^*^	(0.066)	−0.100^*^	(0.055)	−0.174^*^^*^^*^	(0.057)	−0.156^*^^*^	(0.061)	−0.160^*^^*^^*^	(0.061)
Belongs to religion	−0.395^*^^*^^*^	(0.051)			−0.166^*^^*^^*^	(0.064)	−0.127^*^^*^	(0.053)	−0.107^*^^*^	(0.052)	−0.080	(0.053)	−0.092	(0.058)
Subjective financial situation	−0.374^*^^*^^*^	(0.024)			−0.374^*^^*^^*^	(0.024)	−0.046^*^^*^	(0.021)	−0.031	(0.021)	−0.030	(0.021)	−0.031	(0.021)
*Health and well-being*														
Self-reported health	−0.600^*^^*^^*^	(0.023)					−0.144^*^^*^^*^	(0.023)	−0.110^*^^*^^*^	(0.023)	−0.101^*^^*^^*^	(0.023)	−0.100^*^^*^^*^	(0.023)
Mental well-being (GHQ)	0.153^*^^*^^*^	(0.003)					0.111^*^^*^^*^	(0.004)	0.108^*^^*^^*^	(0.004)	0.103^*^^*^^*^	(0.004)	0.103^*^^*^^*^	(0.004)
Has a long-standing illness or disability	0.911^*^^*^^*^	(0.060)					0.089^*^	(0.054)	0.060	(0.053)	0.057	(0.053)	0.061	(0.052)
Life satisfaction	−0.512^*^^*^^*^	(0.014)					−0.200^*^^*^^*^	(0.016)	−0.198^*^^*^^*^	(0.015)	−0.188^*^^*^^*^	(0.015)	−0.187^*^^*^^*^	(0.015)
*Social relationships*														
Hours interacting with friends on social media	−0.006	(0.025)							−0.024	(0.021)	−0.020	(0.020)	−0.017	(0.021)
Going out socially/visiting friends when feeling like it	−0.148^*^^*^^*^	(0.073)							−0.494^*^^*^^*^	(0.062)	−0.495^*^^*^^*^	(0.062)	−0.491^*^^*^^*^	(0.062)
Number of close friends	−0.032^*^^*^^*^	(0.005)							−0.017^*^^*^^*^	(0.004)	−0.016^*^^*^^*^	(0.004)	−0.017^*^^*^^*^	(0.004)
Number of friends similar age	−0.202^*^^*^^*^	(0.028)							−0.054^*^^*^	(0.022)	−0.060^*^^*^^*^	(0.022)	−0.057^*^^*^	(0.024)
Number of friends similar race	−0.059^*^^*^	(0.025)							−0.047^*^^*^	(0.023)	−0.037	(0.023)	−0.039^*^	(0.023)
Number of friends living same area	−0.173^*^^*^^*^	(0.019)							−0.062^*^^*^^*^	(0.016)	−0.043^*^^*^^*^	(0.016)	−0.046^*^^*^^*^	(0.017)
*Community environment*														
Perceived neighbourhood quality	−0.027^*^^*^^*^	(0.005)									−0.009^*^^*^	(0.004)	−0.008^*^^*^	(0.004)
Sense of belonging to neighbourhood	−0.482^*^^*^^*^	(0.023)									−0.085^*^^*^^*^	(0.027)	−0.080^*^^*^^*^	(0.025)
Local friends mean a lot	−0.360^*^^*^^*^	(0.021)									0.018	(0.024)	0.018	(0.024)
Similar to others in neighbourhood	−0.362^*^^*^^*^	(0.020)									−0.048^*^^*^	(0.021)	−0.048^*^^*^	(0.022)
Talk regularly to neighbours	−0.332^*^^*^^*^	(0.018)									−0.055^*^^*^^*^	(0.021)	−0.056^*^^*^^*^	(0.021)
Community type (ref. Blue collar communities): city living	0.369^*^^*^^*^	(0.121)									0.256^*^^*^	(0.105)	0.260^*^^*^	(0.106)
Community type (ref. Blue collar communities): countryside	−0.032	(0.091)									0.123	(0.086)	0.128	(0.088)
Community type (ref. Blue collar communities): prospering suburbs	−0.051	(0.074)									0.139^*^^*^	(0.062)	0.139^*^^*^	(0.064)
Community type (ref. Blue collar communities): constrained by circumstances	0.243^*^^*^	(0.099)									0.054	(0.083)	0.068	(0.081)
Community type (ref. Blue collar communities): typical traits	0.237^*^^*^^*^	(0.080)									0.134^*^^*^	(0.065)	0.138^*^^*^	(0.065)
Community type (ref. Blue collar communities): multicultural	−0.145^*^^*^	(0.072)									0.138^*^	(0.074)	0.149^*^	(0.079)
**Random**:														
Geographic region (local authority district) (S.D.)			0.491	(0.039)	0.436	(0.039)	0.326	(0.032)	0.346	(0.031)	0.328	(0.032)	0.329	(0.032)
Individual level (S.D.)			1.717	(0.016)	1.649	(0.016)	1.393	(0.013)	1.374	(0.013)	1.366	(0.013)	1.362	(0.013)
Observations			6503		6503		6503		6503		6503		6503	
Number of groups			379		379		379		379		379		379	
(ICC)/(VPC)			0.0756		0.0654		0.0520		0.0595		0.0544		0.0551	

Standard errors (se) in parentheses; S.D. = standard deviation; ICC = Intra-class correlation; VPC = Variance partition coefficient.

^*^^*^^*^ *P* < 0.01, ^*^^*^P < 0.05, ^*^P < 0.1.

Results from the final model (Model 5) showed that a range of sociodemographic characteristics were associated with loneliness. Loneliness was lower among older individuals (*b* = −0.025, SE = 0.007), and those of minority ethnic background (*b* = −0.156, SE = 0.061), whereas higher loneliness was found among those of minority sexual orientation (*b* = 0.392, SE = 0.069). Young people in Wales reported lower loneliness than peers in England (*b* = −0.191, SE = 0.115). There was no indication of differences in loneliness between young people living in rural and urban areas. Religious belief and financial situation were not significant predictors of youth loneliness in the final model.

In terms of health and well-being characteristics, higher self-reported health (*b* = −0.101, SE = 0.023), higher life satisfaction (*b* = −0.188, SE = 0.015), and more positive mental well-being (*b* = 0.103, SE = 0.023) were associated with lower loneliness. Having a long-standing illness was associated with higher loneliness, but this effect became non-significant after accounting for social and community factors.

There was strong evidence that social relationships were associated with loneliness. Young people experienced less loneliness if they reported going out with friends (*b* = −0.495, SE = 0.062), having a greater number of close friends (*b* = −0.016, SE = 0.004), a larger number of friends of similar age (*b* = − 0.060, SE = 0.022), and a larger number of friends living in the local area (*b* = −0.043, SE = 0.016). The number of hours spent interacting with friends on social media was not related to loneliness (*b* = −0.020, SE = 0.020).

Community characteristics were also associated with loneliness. Higher levels of perceived neighbourhood quality (*b* = −0.009, SE = 0.004), sense of belonging to the neighbourhood (*b* = −0.085, SE = 0.027), similarity to others in the neighbourhood (*b* = −0.048, SE = 0.021), and talking to neighbours (*b* = −0.055, SE = 0.021) was associated with less loneliness. Reporting that one’s friends in the local area meant a lot was not associated with loneliness (*b* = 0.018, SE = 0.024). Results also indicated that loneliness was more likely in certain types of communities compared to others. Specifically, compared to young people living in communities defined by the Office of National Statistics as ‘blue collar communities’, loneliness was higher among those living in communities categorized as ‘city living’ (*b* = 0.256, SE = 0.105), ‘prospering suburbs’ (*b* = 0.139, SE = 0.062), ‘typical traits’ (*b* = 0.134, SE = 0.065) and ‘multicultural’ (*b* = 0.138, SE = 0.074).

An alternative final model (Model 6), in which sexual orientation was modelled categorically, demonstrated that loneliness was higher among young people who reported ‘other’ sexual orientation (*b* = 0.820, SE = 0.174), followed by those who reported being gay or lesbian (*b* = 0.404, SE = 0.137) and bisexual individuals (*b* = 0.309, SE = 0.085). Using a categorical measure of sexual identity did not substantially alter the significance of other parameters in the model.

Lastly, results from the random-slopes models demonstrated that the effect of gender, minority sexual orientation and minority ethnic background differed across geographic region. Specifically, the between-LA district variation in loneliness was greater for young women, people of minority ethnic background and young people of minority sexual orientation (see Table A1 in Appendix 1).

## Discussion

### Main findings of this study

The aim of the current study was to determine social ecological predictors of loneliness, and the extent to which geographic region may account for differences in loneliness among young people in the UK. Through a multilevel modelling approach, the study found that geographic region accounted for 5–8% of the variation in loneliness. Additionally, the effect of key sociodemographic predictors of loneliness (e.g. gender, sexual orientation and ethnic minority status) were found to differ across geographic regions. For example, in some regions, identifying as a sexual minority mattered more for loneliness than in others, suggesting that there may be place-based differences in the experiences of young people who identify as being of minority sexual orientation.

### What is already known on this topic

A number of our findings support what is already known in relation to youth loneliness. For example, we found no difference in rates of loneliness between young people resident in rural or urban areas.^15^ We found that being from an ethnic minority background was associated with reduced loneliness, which replicated recent findings,^35^ although other research contends that ethnic minority status may increase loneliness because of discrimination.^36^^,^^37^ In support of existing findings^15^^,^^38^ we identified that community environment was influential in youth loneliness, as were factors related to well-being.

### What this study adds

Findings from our study highlight modifiable social and community factors related to loneliness, as well as individual vulnerabilities. For example, we found that sexual orientation was an important predictor of loneliness, and uncovered differences in loneliness across categories of sexual minority identity. LGBTQI+ youth are more likely to experience family rejection, bullying and violence compared to heterosexual or cis-gender peers^39^ and our findings highlight an additional risk of loneliness for this group.

Extant evidence indicates that young people with chronic illness are more likely to be lonely than peers without such illness,^24^^,^^40^ but that research has generally not accounted for broader social ecological factors. Our results show that although having a long-term illness was initially a significant predictor of loneliness, the relationship dissipated after controlling for social and community factors, suggesting that strong bonds with peers and community are important protective factors for preventing loneliness.

Additionally, young people who reported higher perceived neighbourhood quality and felt a greater sense of belonging to their communities were less likely to be lonely. This highlights the importance of developing interventions that promote involvement in the community. Social prescribing has been shown to reduce loneliness in adults^41^^,^^42^ and may be effective in increasing community engagement for young people. Alternatively, interventions at the community level should focus on developing more inclusive communities.

Our findings add to the debate about the role of social media use in youth loneliness. Several studies report a link between increased social media use and loneliness,^43–45^ whereas others report no such association^46^ or that results vary based on type of social media platform.^47^ Our study found that social media use was not a predictor of youth loneliness, while contrastingly, face to face contact (e.g. going out with friends) was associated with reduced loneliness.

Finally, we found some evidence that geographic region may account for a portion of the differences in loneliness in the sample, even after taking individual characteristics into account. Further, we found that the effect of some factors, such as ethnicity and sexual orientation, differed between regions, indicating critical place-based differences in experiences of loneliness.

### Limitations of this study

We used cross-sectional data, thus we cannot infer causality. However, our aim was simply to identify social–ecological risk factors for loneliness, therefore causality was not essential in identifying those at greater risk of loneliness. Additionally, although we were able to identify that loneliness among young people varied depending on geographic region, we do not have region-specific data (e.g. deprivation of region) to explain those differences. However, as LAs may represent a very large area, region-specific data (such as deprivation) is likely to mask variability of individual circumstances (e.g. neighbourhood deprivation versus region deprivation). Relatedly, it is crucial to note that the LA district data used to assess differences in loneliness based on geographic region is heterogeneous in nature. Our data include LA districts with populations ranging in size from 2224 (Isles of Scilly) to 1 141 816 (Birmingham). Given this heterogeneity, and the likely variability of individual circumstances of young people in each region, our evidence regarding geographical differences in loneliness may need to be interpreted with caution. Yet, our study does provide some evidence of geographic differences in loneliness, and provides a compelling argument for future research to use finely grained measures of place to more fully investigate geographic differences in youth loneliness.

## Conclusions

This study provides novel insight into the key social ecological factors associated with loneliness in young people, as well as new evidence on the role of geographic region. The findings are particularly valuable in relation to the development of targeted public health interventions, as results identify key individual vulnerabilities to loneliness, such as sexual orientation and mental well-being, which must be acknowledged when designing interventions. Additionally, results indicate differences in loneliness across regions. As such, it may be more effective to tackle loneliness at a more local level, rather than via broader, less nuanced national approaches, with national strategies taking into account the important role of local conditions and solutions.

## Data availability

The data underlying this article are publicly available in the UK Data Service, Understanding Society, Waves 1-10, at http://doi.org/10.5255/UKDA-SN-6614-14. Local authority district data requires special licence access, available from the UK Data Service, http://doi.org/10.5255/UKDA-SN-6666-12.

## Supplementary Material

Marquez_et_al_2021_revision_appendix_01-11-2021_fdab402

## Notes

**Dr J. Marquez**, ESRC Postdoctoral Fellow.

**Dr C. Goodfellow**, Postdoctoral Research Assistant.

**Ms. D. Hardoon**, Head of Evidence and Analysis

**Dr J. Inchley**, Reader.

**Prof. A. Leyland**, Associate Director (MRC/CSO Social and Public Health Sciences Unit).

**Prof. P. Qualter**, Professor of Psychology for Education.

**Prof. S. A. Simpson**, Professor of Behavioural Sciences and Health.

**Dr E. Long**, MRC Skills Development Fellow.
