# Supplementary material for: Loneliness in young people: a multilevel exploration of social ecological influences and geographic variation
Source: J Public Health (Oxf). 2022 Jan 7;45(1):109–17. doi: 10.1093/pubmed/fdab402 (PMC10017088; doi:10.1093/pubmed/fdab402)
Supplement: Marquez_et_al_2021_revision_appendix_01-11-2021_fdab402 [file marquez_et_al_2021_revision_appendix_01-11-2021_fdab402.docx]

**Appendix 1.**

Table A1. Multilevel models with random effects

|  |  | Model 1 | |  | Model 2 | |  | Model 3 | |
| --- | --- | --- | --- | --- | --- | --- | --- | --- | --- |
|  |  | (gender random effect) | |  | (sexual orientation random effect) | |  | (minority ethnic group random effect) | |
| Variables |  | b | SE |  | b | SE |  | b | SE |
| *Fixed:* |  |  |  |  |  |  |  |  |  |
| Constant |  | 4.113*** | (0.271) |  | 4.090*** | (0.269) |  | 4.145*** | (0.272) |
| Age - 16 to 24 |  | -0.025*** | (0.007) |  | -0.025*** | (0.007) |  | -0.025*** | (0.007) |
| Gender |  | 0.017 | (0.048) |  | -0.024 | (0.039) |  | -0.023 | (0.039) |
| Sexual orientation (binary): not heterosexual |  | 0.408*** | (0.069) |  | 0.402*** | (0.096) |  | 0.394*** | (0.069) |
| Country (ref. England): Wales |  | -0.156 | (0.118) |  | -0.179 | (0.115) |  | -0.193* | (0.114) |
| Country (ref. England): Scotland |  | 0.005 | (0.105) |  | -0.043 | (0.102) |  | -0.054 | (0.101) |
| Country (ref. England): Northern Ireland |  | 0.060 | (0.128) |  | 0.093 | (0.128) |  | 0.084 | (0.129) |
| Living in urban or rural area |  | -0.006 | (0.067) |  | 0.014 | (0.067) |  | 0.009 | (0.067) |
| Minority ethnic group (non-white British) |  | -0.148** | (0.061) |  | -0.158*** | (0.060) |  | -0.142** | (0.068) |
| Belongs to religion |  | -0.085 | (0.053) |  | -0.088* | (0.053) |  | -0.079 | (0.053) |
| Subjective financial situation |  | -0.030 | (0.021) |  | -0.027 | (0.020) |  | -0.029 | (0.021) |
| Self-reported health |  | -0.101*** | (0.023) |  | -0.099*** | (0.023) |  | -0.102*** | (0.023) |
| Mental wellbeing (GHQ) |  | 0.103*** | (0.004) |  | 0.105*** | (0.004) |  | 0.103*** | (0.004) |
| Has a long-standing illness or disability |  | 0.058 | (0.053) |  | 0.046 | (0.053) |  | 0.059 | (0.053) |
| Life satisfaction |  | -0.190*** | (0.016) |  | -0.192*** | (0.016) |  | -0.188*** | (0.016) |
| Hours interacting with friends on social media |  | -0.017 | (0.021) |  | -0.018 | (0.020) |  | -0.019 | (0.020) |
| Going out socially/visiting friends when feeling like it |  | -0.494*** | (0.062) |  | -0.501*** | (0.062) |  | -0.490*** | (0.062) |
| Number of close friends |  | -0.017*** | (0.004) |  | -0.017*** | (0.004) |  | -0.016*** | (0.004) |
| Number of friends similar age |  | -0.059*** | (0.023) |  | -0.055** | (0.022) |  | -0.061*** | (0.022) |
| Number of friends similar race |  | -0.035 | (0.023) |  | -0.042* | (0.0233) |  | -0.034 | (0.023) |
| Number of friends living same area |  | -0.047*** | (0.016) |  | -0.042*** | (0.016) |  | -0.044*** | (0.016) |
| Perceived neighbourhood quality |  | -0.009** | (0.004) |  | -0.009** | (0.004) |  | -0.009** | (0.004) |
| Sense of belonging to neighbourhood |  | -0.079*** | (0.027) |  | -0.068** | (0.027) |  | -0.084*** | (0.027) |
| Local friends mean a lot |  | 0.015 | (0.024) |  | 0.020 | (0.024) |  | 0.018 | (0.024) |
| Similar to others in neighbourhood |  | -0.045** | (0.021) |  | -0.051** | (0.021) |  | -0.048** | (0.021) |
| Talk regularly to neighbourhood |  | -0.052** | (0.021) |  | -0.060*** | (0.021) |  | -0.054** | (0.021) |
| Community type (ref. Blue collar): city living |  | 0.263** | (0.104) |  | 0.323*** | (0.106) |  | 0.233** | (0.105) |
| Community type (ref. Blue collar): countryside |  | 0.131 | (0.086) |  | 0.135 | (0.087) |  | 0.116 | (0.086) |
| Community type (ref. Blue collar): prospering suburbs |  | 0.148** | (0.062) |  | 0.156** | (0.062) |  | 0.131** | (0.063) |
| Community type (ref. Blue collar): constrained by circumstances |  | 0.039 | (0.083) |  | 0.084 | (0.082) |  | 0.057 | (0.083) |
| Community type (ref. Blue collar): typical traits |  | 0.136** | (0.065) |  | 0.157** | (0.065) |  | 0.132** | (0.065) |
| Community type (ref. Blue collar): multicultural |  | 0.143* | (0.074) |  | 0.161** | (0.074) |  | 0.126* | (0.077) |
| *Random:* |  |  |  |  |  |  |  |  |  |
| Geographic region level (S.D., 95% C.I., S.E.) |  | 0.274 | |  | 0.321 | |  | 0.317 | |
|  |  | (0.209 - 0.360) | |  | (0.264 - 0.391) | |  | (0.258 - 0.390) | |
|  |  | (0.038) | |  | (0.032) | |  | (0.033) | |
| Gender random effect (S.D., 95% C.I., S.E.) |  | 0.459 | |  |  |  |  |  |  |
|  |  | (0.377 - 0.557) | |  |  |  |  |  |  |
|  |  | (0.046) | |  |  |  |  |  |  |
| Sexual orientation random effect (S.D., 95% C.I., S.E.) |  |  |  |  | 0.859 | |  |  |  |
|  |  |  |  |  | (0.691 - 1.067) | |  |  |  |
|  |  |  |  |  | (0.095) | |  |  |  |
| Minority ethnic group random effect (S.D., 95% C.I., S.E.) |  |  |  |  |  |  |  | 0.316 | |
|  |  |  |  |  |  |  |  | (0.187 - 0.532) | |
|  |  |  |  |  |  |  |  | (0.084) | |
| Individual level (S.D., 95% C.I., S.E.) |  | 1.345 | |  | 1.346 | |  | 1.361 | |
|  |  | (1.318 - 1.372) | |  | (1.320 - 1.372) | |  | (1.335 - 1.388) | |
|  |  | (0.014) | |  | (0.013) | |  | (0.013) | |
| Observations |  | 6,503 | |  | 6,503 | |  | 6,503 | |
| Number of groups |  | 379 | |  | 379 | |  | 379 | |
| Standard errors in parentheses; *** p<0.01, ** p<0.05, * p<0.1  S.D. = Standard Deviation; S.E. = Standard Error; C.I. = Confidence Interval | | | | | | | | | |

Table A1. Multilevel models with random effects (continuation)

|  |  | Model 4 | |  | Model 5 | |  | Model 6 | |
| --- | --- | --- | --- | --- | --- | --- | --- | --- | --- |
|  |  | (age random effect) | |  | (subjective financial situation random effect) | |  | (belonging to neighbourhood random effect) | |
| Variables |  | b | SE |  | b | SE |  | b | SE |
| *Fixed:* |  |  |  |  |  |  |  |  |  |
| Constant |  | 4.157*** | (0.271) |  | 4.159*** | (0.271) |  | 4.158*** | (0.271) |
| Age - 16 to 24 |  | -0.025*** | (0.007) |  | -0.025*** | (0.007) |  | -0.025*** | (0.007) |
| Gender |  | -0.023 | (0.037) |  | -0.023 | (0.039) |  | -0.023 | (0.039) |
| Sexual orientation (binary): not heterosexual |  | 0.393*** | (0.069) |  | 0.393*** | (0.069) |  | 0.392*** | (0.069) |
| Country (ref. England): Wales |  | -0.192* | (0.116) |  | -0.191 | (0.117) |  | -0.191* | (0.115) |
| Country (ref. England): Scotland |  | -0.050 | (0.102) |  | -0.055 | (0.104) |  | -0.051 | (0.102) |
| Country (ref. England): Northern Ireland |  | 0.073 | (0.129) |  | 0.060 | (0.131) |  | 0.073 | (0.129) |
| Living in urban or rural area |  | 0.008 | (0.069) |  | 0.004 | (0.067) |  | 0.008 | (0.067) |
| Minority ethnic group (non-white British) |  | -0.156** | (0.061) |  | -0.161*** | (0.061) |  | -0.156** | (0.061) |
| Belongs to religion |  | -0.080 | (0.053) |  | -0.079 | (0.053) |  | -0.080 | (0.053) |
| Subjective financial situation |  | -0.030 | (0.021) |  | -0.029 | (0.021) |  | -0.030 | (0.021) |
| Self-reported health |  | -0.101*** | (0.023) |  | -0.101*** | (0.023) |  | -0.101*** | (0.023) |
| Mental wellbeing (GHQ) |  | 0.103*** | (0.004) |  | 0.103*** | (0.004) |  | 0.103*** | (0.004) |
| Has a long-standing illness or disability |  | 0.057 | (0.052) |  | 0.056 | (0.052) |  | 0.057 | (0.052) |
| Life satisfaction |  | -0.188*** | (0.015) |  | -0.188*** | (0.015) |  | -0.188*** | (0.015) |
| Hours interacting with friends on social media |  | -0.020 | (0.020) |  | -0.020 | (0.020) |  | -0.020 | (0.020) |
| Going out socially/visiting friends when feeling like it |  | -0.495*** | (0.062) |  | -0.497*** | (0.062) |  | -0.495*** | (0.062) |
| Number of close friends |  | -0.016*** | (0.004) |  | -0.016*** | (0.004) |  | -0.016*** | (0.004) |
| Number of friends similar age |  | -0.060*** | (0.022) |  | -0.061*** | (0.022) |  | -0.060*** | (0.022) |
| Number of friends similar race |  | -0.037 | (0.023) |  | -0.037 | (0.023) |  | -0.037 | (0.023) |
| Number of friends living same area |  | -0.043*** | (0.016) |  | -0.043*** | (0.016) |  | -0.043*** | (0.016) |
| Perceived neighbourhood quality |  | -0.009** | (0.004) |  | -0.009** | (0.004) |  | -0.009** | (0.004) |
| Sense of belonging to neighbourhood |  | -0.084*** | (0.027) |  | -0.083*** | (0.027) |  | -0.085*** | (0.027) |
| Local friends mean a lot |  | 0.018 | (0.024) |  | 0.017 | (0.024) |  | 0.018 | (0.024) |
| Similar to others in neighbourhood |  | -0.048** | (0.021) |  | -0.049** | (0.021) |  | -0.048** | (0.021) |
| Talk regularly to neighbourhood |  | -0.055*** | (0.021) |  | -0.054*** | (0.021) |  | -0.055*** | (0.021) |
| Community type (ref. Blue collar): city living |  | 0.256** | (0.105) |  | 0.257** | (0.105) |  | 0.256** | (0.105) |
| Community type (ref. Blue collar): countryside |  | 0.122 | (0.086) |  | 0.124 | (0.087) |  | 0.123 | (0.086) |
| Community type (ref. Blue collar): prospering suburbs |  | 0.139** | (0.062) |  | 0.138** | (0.063) |  | 0.139** | (0.062) |
| Community type (ref. Blue collar): constrained by circumstances |  | 0.054 | (0.083) |  | 0.051 | (0.083) |  | 0.054 | (0.083) |
| Community type (ref. Blue collar): typical traits |  | 0.134** | (0.065) |  | 0.131** | (0.065) |  | 0.134** | (0.065) |
| Community type (ref. Blue collar): multicultural |  | 0.138* | (0.074) |  | 0.135* | (0.075) |  | 0.138* | (0.074) |
| *Random:* |  |  |  |  |  |  |  |  |  |
| Geographic region level (S.D., 95% C.I., S.E.) |  | 0.324 | |  | 0.321 | |  | 0.317 | |
|  |  | (0.241 - 0.437) | |  | (0.264 - 0.391) | |  | (0.258 - 0.390) | |
|  |  | (0.049) | |  | (0.032) | |  | (0.033) | |
| Age random effect (S.D., 95% C.I., S.E.) |  | 0.000 | |  |  |  |  |  |  |
|  |  | (9.42e-87 - 2.47e+77) | |  |  |  |  |  |  |
|  |  | (0.005) | |  |  |  |  |  |  |
| Subjective financial situation random effect (S.D., 95% C.I., S.E.) |  |  |  |  | 0.049 | |  |  |  |
|  |  |  |  |  | (0.025 - 0.096) | |  |  |  |
|  |  |  |  |  | (0.017) | |  |  |  |
| Sense of belonging to neighbourhood random effect (S.D., 95% C.I., S.E.) |  |  |  |  |  |  |  | 1.40e-07 | |
|  |  |  |  |  |  |  |  | (8.37e-59 - 2.33e+44) | |
|  |  |  |  |  |  |  |  | (8.41e-06) | |
| Individual level (S.D., 95% C.I., S.E.) |  | 1.366 | |  | 1.346 | |  | 1.366 | |
|  |  | (1.341 - 1.392) | |  | (1.320 - 1.372) | | 374 | (1.341 - 1.392) | |
|  |  | 0.013 | |  | (0.013) | |  | (0.013) | |
| Observations |  | 6,503 | |  | 6,503 | |  | 6,503 | |
| Number of groups |  | 379 | |  | 379 | |  | 379 | |
| Standard errors in parentheses; *** p<0.01, ** p<0.05, * p<0.1  S.D. = Standard Deviation; S.E. = Standard Error; C.I. = Confidence Interval | | | | | | | | | |
